# Evolutionary rearrangements of X chromosomes in voles (Arvicolinae, Rodentia)

**DOI:** 10.1038/s41598-020-70226-4

**Published:** 2020-08-06

**Authors:** Svetlana A. Romanenko, Yulia E. Fedorova, Natalya A. Serdyukova, Marco Zaccaroni, Roscoe Stanyon, Alexander S. Graphodatsky

**Affiliations:** 1 Institute of Molecular and Cellular Biology, SB RAS, Novosibirsk, Russia; 2 Novosibirsk State University, Novosibirsk, Russia; 3 Department of Biology, University of Florence, Florence, Italy

**Keywords:** Genetics, Cytogenetics

## Abstract

Euchromatic segments of the X chromosomes of placental mammals are the most conservative elements of the karyotype, only rarely subjected to either inter- or intrachromosomal rearrangements. Here, using microdissection-derived set of region-specific probes of *Terricola savii* we detailed the evolutionary rearrangements found in X chromosomes in 20 vole species (Arvicolinae, Rodentia). We show that the evolution of X chromosomes in this taxon was accompanied by multiple para- and pericentric inversions and centromere shifts. The contribution of intrachromosomal rearrangements to the karyotype evolution of Arvicolinae species was approximately equivalent in both the separate autosomal conserved segments and the X chromosomes. Intrachromosmal rearrangements and structural reorganization of the X chromosomes was likely accompanied by an accumulation, distribution, and evolution of repeated sequences.

## Introduction

Most eutherians have two sex chromosomes (gonosomes)—X and Y. The gonosomes are thought to have emerged from a pair of autosomes with the advent of the sex-determining gene^1^. Between 166–105 million years ago a number of Robertsonian translocations between the sex chromosomes and autosomes occurred^2^. The gonosomes soon diverged and became heteromorphic due to the absence of recombination in all but a small pseudoautosomal region on both chromosomes. Over the last 70 years cytogenetists have documented the morphology, centromere position, heterochromatin content and distribution of X and Y chromosomes of various mammalian species. One important conclusion was that the X chromosomes were often highly conserved even between distantly related species^3^. This conservatism of the X chromosome even in phylogenetically distant mammals, such as humans, pigs, horses, dogs, and cats, was amply confirmed by the study of genetic marker order^4^. Comparative cytogenetic studies often show that the X chromosome remains conserved even when almost all autosomes are highly rearranged, for example, dogs have highly rearranged autosomes compared to other carnivores, but the X chromosome is conserved^5,6^. It is well appreciated that rodents are generally characterized by highly rearranged genomes, but nonetheless many rodents (beavers, squirrels) have a conserved X chromosome^7^. It is thought that the X chromosome is conserved as a result of a dose compensation mechanism that imposes evolutionary restrictions on rearrangements^8,9^.

However, there are well known cases when the X chromosome, both with respect to gene content and marker order^10^, is not conserved. The X chromosomes of a significant number of mouse-like rodents (Myomorpha) and cetartiodactyls are clearly rearranged and subject to both intrachromosomal and interchromosomal rearrangements^11-14^. The reasons why some taxa escape X chromosome conservatism are not clear.

Myomorpha is the largest placental suborder and it is characterized by high rates of karyotypic evolution. The mouse-like rodents have the highest number of species among mammals with rearranged sex chromosomes^15^. It appears that sex chromosomes in these species are most often subject to translocation with autosomes and the Y chromosome is often lost. Frequent variations in centromere positions, even in closely related species, indicate that pericentric inversions and/or the emergence of evolutionarily new centromeres (ENC) are common. Cases of the emergence of ENC on gonosomes have been confirmed for species of the genus *Tokudaia*^16^. *Tokudaia tokunoshimensis* has the same localization of centromere as *Rattus norvegicus*, while *T. osimensis* has an ENC, that presumably appeared after the divergence of the genus *Tokudaia* and their common ancestor with *R. norvegicus*^16^. Some populations of *Microtus agrestis* have a so-called “Lu-Y” chromosome formed due to pericentric inversion of the Y chromosome^17^.

The huge variety of sex chromosome systems described for myomorphs makes them unique among mammals and even rodents from other suborders. Moreover, autosomal sets of mouse-like rodents also underwent a mega reorganization during evolution due to numerous intra- and interchromosomal rearrangements^18,19^.

Recently, it was shown that autosomal syntenic blocks in Arvicolinae karyotypes were subjected to multiple evolutionary rearrangements. Apparently, the number of intrachromosomal rearrangements exceeded interchromosomal rearrangements^20,21^. It is important to note that the autosomes of voles often have some amount of pericentromeric heterochromatin. The accumulation of heterochromatin and duplications of tandem repeats can significantly affect the morphology of sex chromosomes in mouse-like rodents. Some arvicolines (*M. agrestis, M. cabrerae, M. chrotorrhinus, M. epiroticus,* and *M. transcaspicus*) have so-called "giant" sex chromosomes, representing up to 20% of the genome. Variation in length and morphology (from acrocentric to metacentric) of the gonosomes, in this case, could be caused by the inclusion of inhomogeneous heterochromatic blocks. Previously it has been shown that such blocks were capable of forming whole heterochromatic arms of chromosomes^22^. C-banding reveals heterochromatic blocks that make up more than half the length of the X chromosome in many species. In addition, some species exhibit hypervariability in the amount and distribution of heterochromatin (e.g. *Lasiopodomys mandarinus*^23^).

Unfortunately, the evolution of the X chromosomes of rodents as well as other mammals is not well understood. Previously, X chromosomes of only five species of the genus *Microtus* (*M. arvalis, M. kirgisorum, M. rossiaemeridionalis, M. transcaspicus, M. agrestis*) were investigated using region-specific probes of the species *M. rossiaemeridionalis*. The study revealed differences in X chromosomes resulted from inversions or intrachromosomal translocations (exchange of chromosomal segments within the same chromosome). The authors reconstructed the possible evolution of the X chromosome during karyotype divergence, underlying the presence of repeated sequences and their possible participation in intrachromosomal rearrangements^24^.

Here, on a large sample of arvicoline species, we report the stability of the euchromatic regions of the X chromosomes and show a momentous contribution of intrachromosomal rearrangements and accumulation of repeated sequences to the evolution of their X chromosomes.

## Methods

### Compliance with ethical standards

All applicable international, national, and/or institutional guidelines for the care and use of animals were followed. All experiments were approved by the Ethics Committee on Animal and Human Research of the Institute of Molecular and Cellular Biology, Siberian Branch of the Russian Academy of Sciences, Russia (order No. 32 of May 5, 2017). This article does not contain any studies with human participants performed by any of the authors.

### Species sampled

We used chromosome suspensions obtained from cell lines in the Laboratory of Animal Cytogenetics, the IMCB SB RAS, Russia. All cell lines were retrieved from the IMCB SB RAS cell bank (“The general collection of cell cultures”, No 0310-2016-0002). The list of species is presented in Table 1: the origin of each sample, the establishment of cell lines, karyotype description for each studied species were previously reported^25-27^.

**Table 1:** List of 20 Arvicolinae species used in the study representing 3 arvicoline tribes and 10 genera.

Tribe	Subtribe	Genus	Subgenus	Species/Subspecies	Abbreviation	Sex	2n	Karyotype description
Arvicolini	*Arvicolina*	*Arvicola*		*A. amphibius (*= *terrestris)*	AAMP	♀	36	^27^
	*Microtina*	*Alexandromys*		*A. evoronensis*	AEVO	♂	36	–
				*A. maximowiczii*	AMAX	♂	44	–
				*A. mujanensis*	AMUJ	♂	38	^18^
		*Blanfordimys*		*B. afghanus*^*¶*^	BAFG	♀	58	–
				*B. juldaschi*^*¶¶*^	BJUL	♂	54	–
		*Chionomys*		*C. gud*	CGUD	♂	54	–
		*Lasiopodomys*	*Lasiopodomys*	*L. brandtii*	LBRA	♂	34	^28^
			*Stenocranius*	L. gregalis*	LGRE	♂	36	^26,28^
		*Microtus*	*Microtus*	*M. arvalis*	MARV	♂	46	^26^
				*M. rossiaemeridionalis (*= *levis)*	MROS	♂	54	^26^
			*Sumeriomys*	*M. dogramacii*^*¶*^	MDOG	♀	48	^26^
				*M. guentheri*^*¶*^	MGUG	♂	54	^26^
				*M. schidlovskii*^*¶*^	MSCH	♂	60	–
		*Terricola*		*T. daghestanicus*	TDAG	♀	54	^26^
				*T. majori*	TMAJ	♀	54	–
				*T. savii*^*¶*^	TSAV	♀	54	–
Ellobiini		*Ellobius*	*Ellobius*	*E. talpinus*	ETAL	♂	54	^29,30^
Myodini		*Alticola*	*Alticola*	*A. tuvinicus*	ATUV	♂	56	–
		*Myodes (*= *Clethrionomys)*		*M. (*= *C. rutilus)*	MRUT	♂	56	^27^

Overall species names here follow the latest checklist “The mammals of Russia: a taxonomic and geographic reference”^31^, names in parentheses are outdated or follow other sources. ^*¶*^—the systematic status of the species defined by^32^. ^*¶¶*^—the systematic status of the species defined by^33^. *—belonged to *Microtus* genus and *Stenocranius* subgenus in^34^. **—the species is listed as *M. maximowizcii* in^34^. Minus signs indicate that the species have not been involved in comparative studies with painting probes yet or specimens with a different from previously published chromosome number were investigated here.

### Chromosome preparation and chromosome staining

Chromosome suspensions were obtained from cell lines according to earlier published protocols^35,36^. G-banding was performed on chromosomes of all species prior to FISH using the standard trypsin/Giemsa treatment procedure^37^. C-banding was performed as described previously^36,38^.

### Microdissection, probe amplification, and labeling

We decided to generate microdissected probes from *Terricola savii* for a number of reasons. It is known that *T. savii* populations differ for the morphology of the X chromosome^39^. Here we utilized individuals from Imola, Italy. The X chromosome was clearly distinguishable in metaphases plates because it is the only metacentric in the karyotype . Further, we concluded that that the X chromosome of *T. savii* individuals from Imola do not have large C-positive blocks. The X-chromosome is small even compared to the X chromosomes of other arvicoline species known not to have large additional heterochromatic blocks. Addtionally, previously published reports on differential staining of chromosomes of *T. savii* confirmed that the metacentric form of this chromosome does not have large heterochromatic blocks^39^.

**Figure 1:**
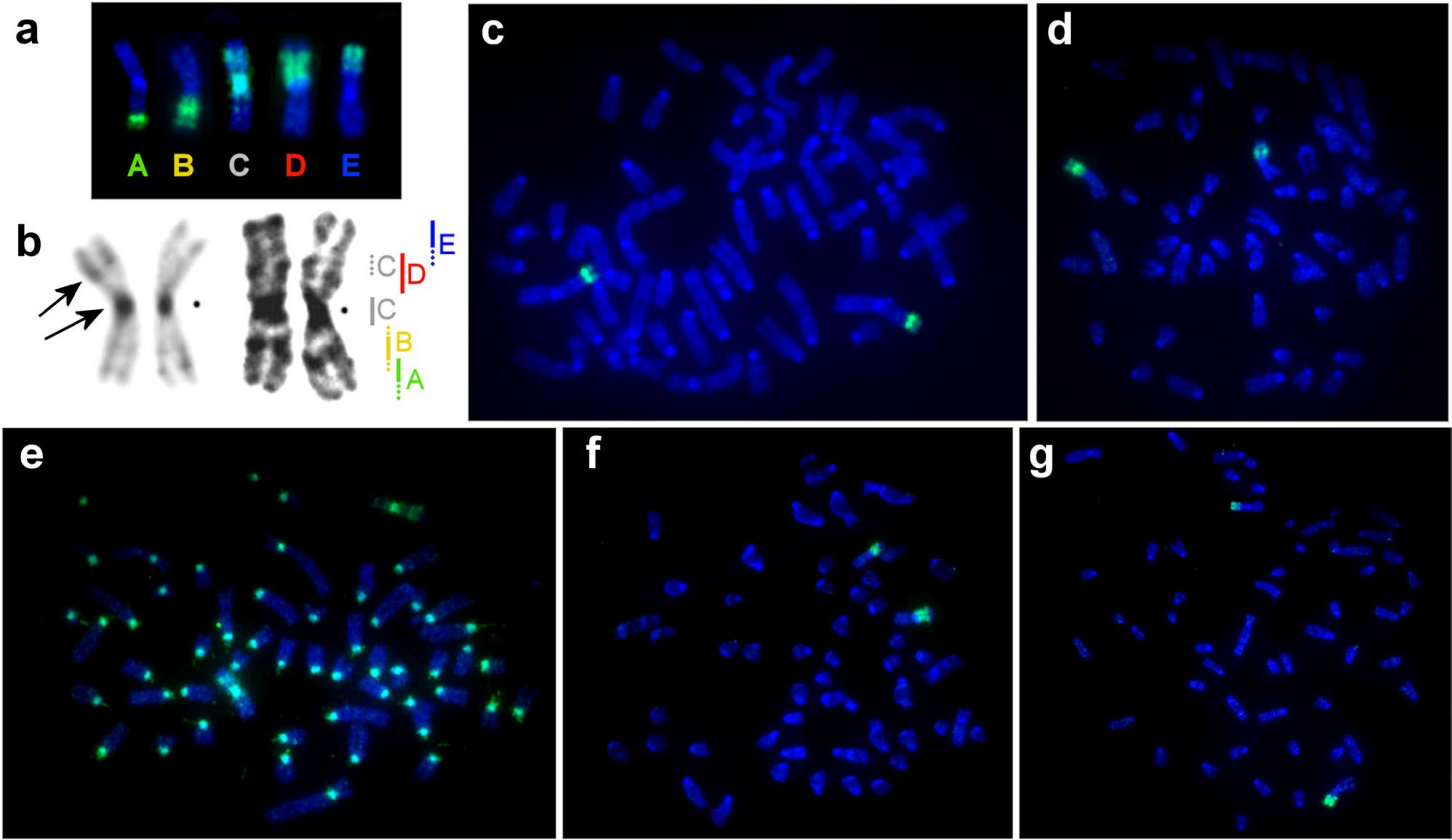
Metaphase chromosomes of *T. savii.* (**a**) Localization of microdissection-derived probes A, B, C, D, and E on *T. savii* DAPI-banded X chromosome, (**b**) *T. savii* X chromosome: C-banding shown on the left, and GTG-banding on the right. Black arrows mark pericentromeric and interstitial heterochromatic regions. Vertical lines indicate the localization of the region-specific probes used in the work. The continuous line indicates the location of the main signal of the probe, the dotted line – the additional signal. Black dots mark the position of centromere. Localization of region-specific probes on *T. savii* chromosomes: (**c**) probe A, (**d**) probe B, (**e**) probe C, (**f**) probe D, (**g**) probe E.

Glass needle-based microdissection was performed as described earlier^40^. Seven copies of each X chromosome region from *T. savii* were collected. Chromosomal DNA was amplified and labeled using WGA kits (Sigma) according to the manufacturer's protocol. In total, we obtained 5 region-specific painting probes covering the whole X chromosome of *T. savii* (Fig. 1).

### Fluorescence in situ hybridization (FISH)

FISH was performed following previously published protocols^6,41^. Images were captured using VideoTest-FISH software (VideoTesT) with a JenOptic CCD camera mounted on an Olympus BX53 microscope. Hybridization signals were assigned to specific chromosome regions defined by G-banding pattern captured by the CCD camera prior to FISH. All images were processed using Corel Paint Shop Pro X2 and X3 (Corel).

### Data analysis

When analyzing the results, we used a combination of different approaches. First, we identified the most common combinations of the structure of the ancestral X chromosome. Secondly, comparative chromosome painting data were compared with the previously established and published phylogenies of Arvicolinae^42-44^. Here, a comparison with outgroup group at the level of individual genera and tribes was made. The reconstruction of the likely structure of the ancestral arvicoline X chromosome was carried out in accordance with the principles of cladistics: the most likely evolution scenario is the one that includes the smallest number of rearrangements (presence of synapomorphies, avoidance of homoplasies)^45^.

## Results

Using microdissection, a set of 5 region-specific painting DNA-probes, covering the whole X chromosome of the Savi's vole (*T. savii*), was established. To clarify the boundaries of probes localization, fluorescence in situ hybridization of the probes to *T. savii* chromosomes was performed (Fig. 1c–g). It is important to note that additional signals of the probe C were localized on the centromeric regions of all autosomes and in the p-arm of the X chromosome (Fig. 1e). We performed C-banding of *T. savii* chromosomes and found a heterochromatic block in the p-arm of the X chromosome, which corresponded well to the location of this additional signal (Fig. 1b). Also, the probes D and E partially overlapped with this heterochromatic block (Fig. 1a,b).

**Figure 2:**
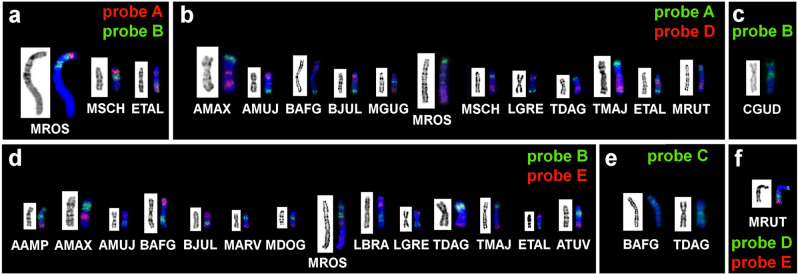
Results of localization of microdissection-derived probes on the chromosomes of some species of voles: (**a**) A (red) and B (green), (**b**) A (green) and D (red), (**c**) B (green), (**d**) B (green) and E (red), (**e**) C (green), (**f**) D (green) and E (red). GTG-banding shown on the left. Abbreviated names of species correspond to Table 1.

The set of probes was used for the comparison of chromosomes of the wide range of Arvicolinae species (Table 1). Hybridization efficiency varied between species, but it was sufficient for probe mapping. The difference in size between the localization areas of the probes in different species might be caused by the amplification of repeated sequences. Examples of fluorescence in situ hybridizations are shown in Figs. 2 and S1.

**Figure 3:**
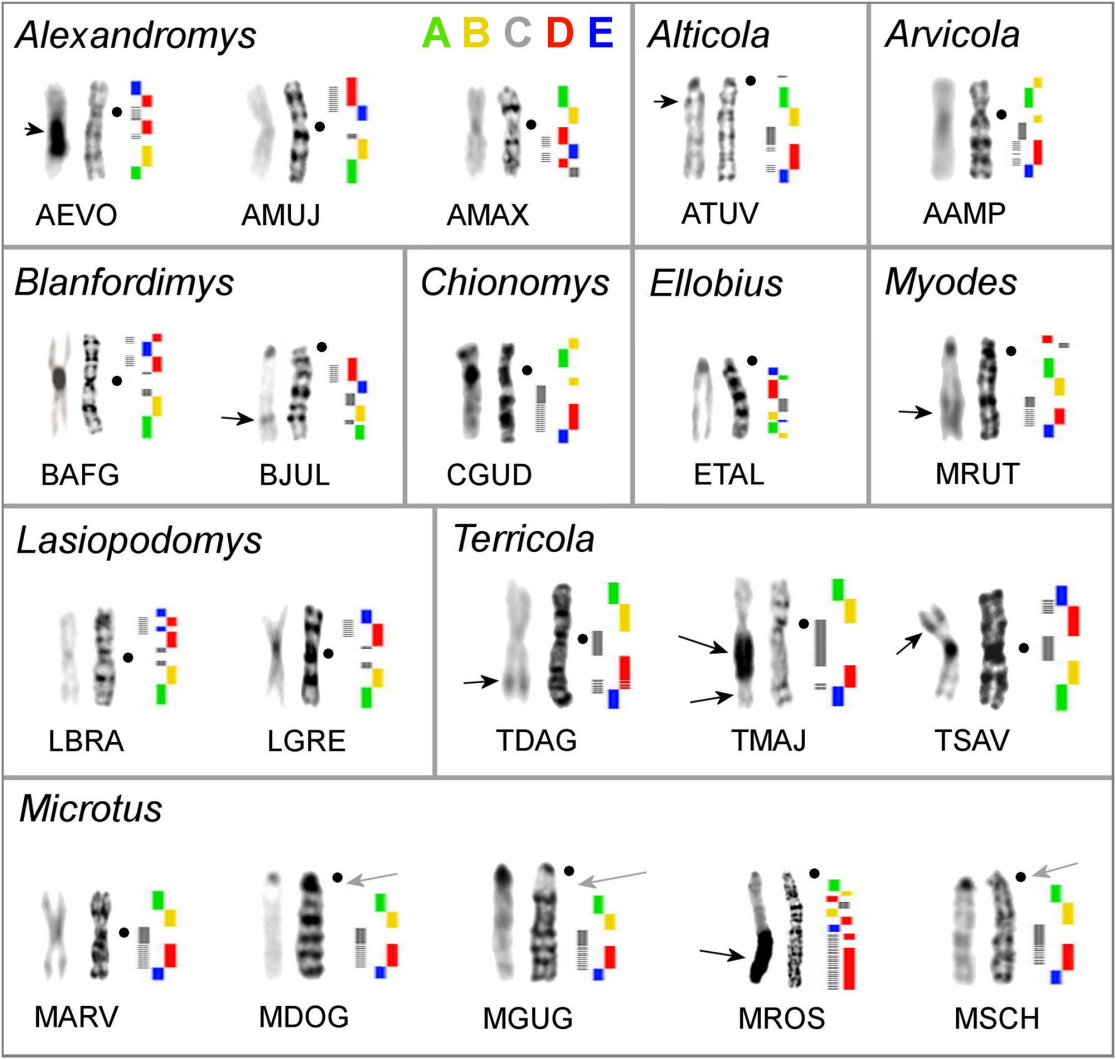
X chromosomes of the investigated arvicoline species. From left to right: C-banding (present data for ATUV*,* BJUL*,* MSCH, TDAG*,* TMAJ, other from previously published works^46–49^), G-banding, probe localization. The continuous line indicates the location of the main signal of the probe, the dotted line—the additional signal. Black dots mark centromere positions. Black arrows mark heterochromatic regions. Grey arrows mark regions that were not labeled by any probes.

In the case of probe C, a clear signal was perceived for most species, however, on chromosomes of five species (*Alexandromys evoronensis, Chionomys gud, L. gregalis, M. arvalis, M. schidlovskii*) the probe had a discrete signal, which made it difficult to establish the boundaries of the hybridization. This probe also did not label centromeres in any of the species, except for those of the *Terricola* group where the probe C had a discrete signal in pericentromeric regions of X chromosomes. Probes D and E, when localized to the chromosomes of some species, had additional signals. Because these additional signals were often covered by the probe C, we assumed that they might also coincide with heterochromatic blocks, as it is with *T. savii*. Moreover, in karyotypes of most species (mostly *Microtus*) probe D had a discrete signal (Fig. 2b). The result of the localization of the full set of probes on the chromosomes of all studied species is presented in Fig. 3.

C-banding of the chromosomes of most of the species used in the study was previously performed^46-50^. Here, in addition to *T. savii*, we carried out C-banding of chromosomes of six more species of voles (*A. mujanensis, Alticola tuvinicus, Blanfordimys juldaschi, M. dogramacii, T. daghestanicus,* and *T. majori*), which allowed to visualise not only centromeric, but interstitial heterochromatic blocks on X chromosomes of four of these species—*A. tuvinicus, B. juldaschi, T. daghestanicus,* and *T. majori* (Fig. 3).

**Figure 4:**
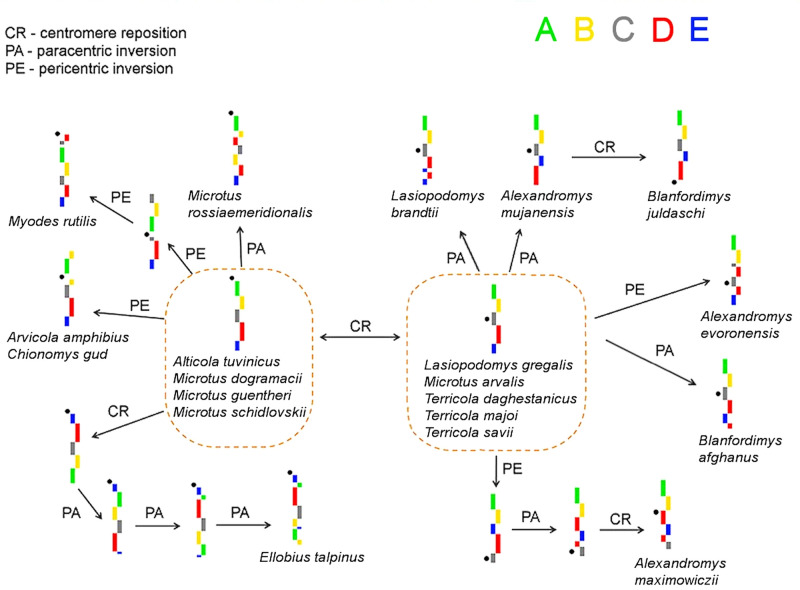
Diagram of rearrangements of X chromosomes in voles. The dotted line circles the presumptive ancestral versions of the X chromosome. Black dots mark centromere positions. The scheme does not reflect variations caused by the number and distribution of repeated sequences. The scheme does not reflect the phylogenetic relationships between species.

The analysis of the obtained patterns of localization of region-specific probes revealed two predominant types of X chromosome configuration differing in centromere position only (Fig. 4). Of the 20 species analyzed, four species had an acrocentric X chromosome, and five had a metacentric X chromosome with the same order of probes. We assumed that one of the morphological types represent a putative ancestral variant of the arvicoline X chromosome. The reconstruction of possible transformation paths that led to the formation of the X chromosomes of modern species of voles was made (Fig. 4).

**Figure 5:**
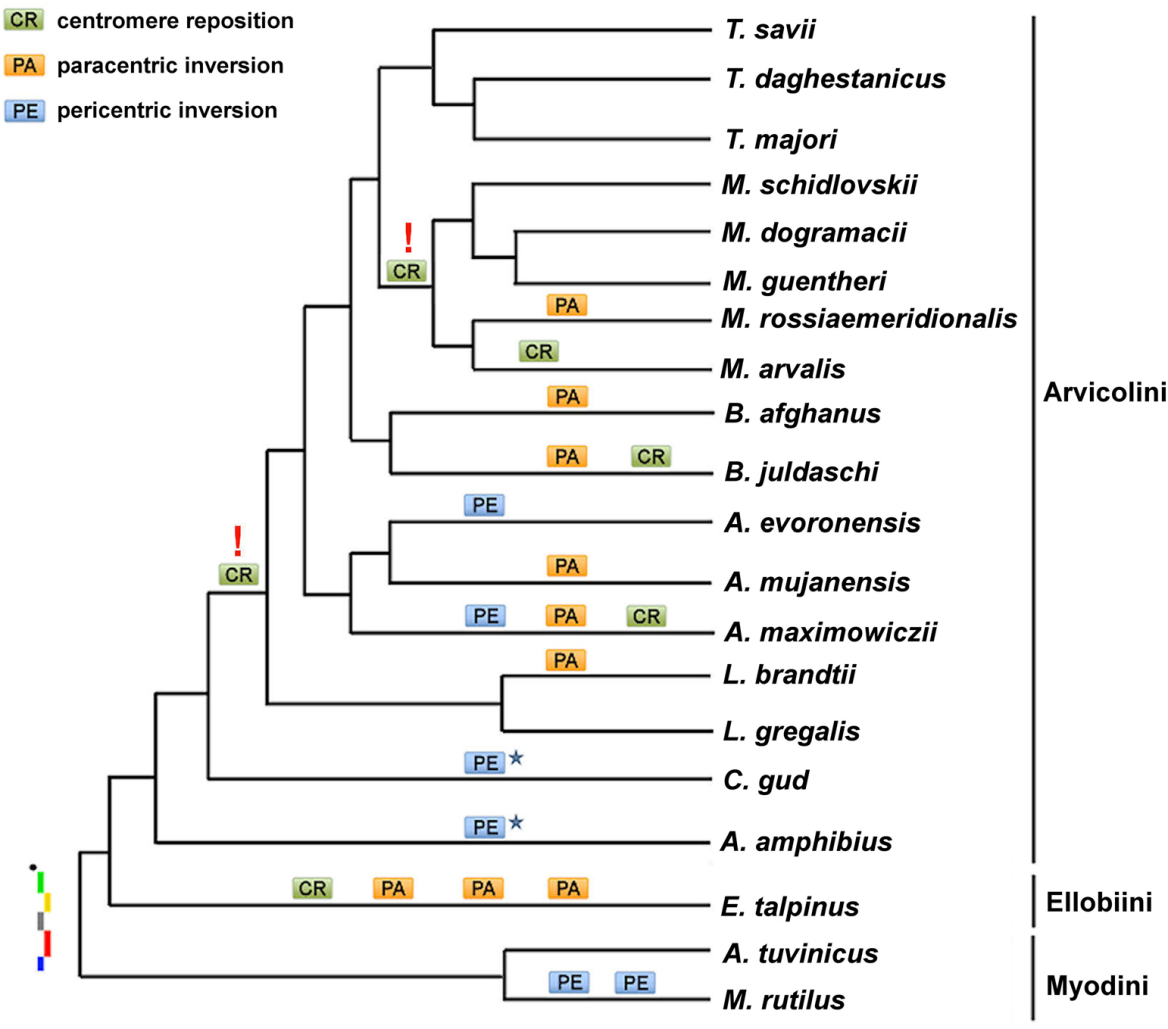
Phylogenetic tree of the Arvicolinae^42–44^ with additions: intrachromosomal rearrangements are indicated above the branch, the alleged ancestral X chromosome is placed at the base of the tree. An asterisk denotes the same pericentric inversions (convergent event). Red exclamation marks indicate the possible places of ambiguous development of the scenario of karyotypic evolution (see text). The tree shows only branching, the relative scale and length of the branches are not informative.

The analysis of the painting data and the previously obtained pattern of phylogenetic relationships in Arvicolinae subfamily suggests that the ancestral X chromosome of the voles was probably acrocentric with probe order from the centromere A-B-C-D-E. To better visualize the results and determine the number and distribution of intrachromosomal rearrangements in different groups of voles, the rearrangements were plotted on a previously published phylogenetic tree of Arvicolinae^42-44^ (Fig. 5).

## Discussion

Arvicolinae is a multi-species and rapidly evolving taxon. Recent molecular studies have clarified phylogenetic relationships in the subfamily, and cytogenetic studies were able to distinguish morphologically similar species and reconstruct the ancestral karyotype of the subfamily based on the analysis of interchromosomal rearrangements^25,26,29,30,51^. It was also shown that vole karyotype evolution was accompanied by intrachromosomal rearrangements: at least three ancestral autosomal conservative segments underwent significant reorganization due to inversions and centromere shifts^20,21^. Recent work has raised questions about the prevalence and importance of intrachromosomal rearrangements in vole karyotype evolution.

### Intrachromosomal rearrangements in the evolution of the X chromosomes of voles

To date, studies of the evolution of vole sex chromosomes were mainly limited to the descriptions of morphology and localization of repeated sequences. However, region-specific X chromosome probes of *M. rossiaemeridionalis* showed that differences in the X chromosomes of five species from the genus *Microtus* could be due to inversions or intrachromosomal translocations^24^.

In this research, having localized the set of region-specific microdissected probes of the Savi's vole on chromosomes of 19 species of voles belonging to different tribes, we assumed that the ancestral X chromosome of voles was acrocentric. This type of X chromosome morphology is concordant with the previously proposed version of the ancestral karyotype of the voles, consisting of 56 acrocentric chromosomes^52^. We were able to map multiple intrachromosomal rearrangements including 9 paracentric inversions, 6 pericentric inversions, and 6 centromere shifts. Although there was no indication of any prevailing type of rearrangements between groups, the results showed that X chromosomes of voles, not only of the genus *Microtus*, frequently undergo intrachromosomal rearrangements. Such high variability in X chromosome morphology generated by intrachromosomal rearrangements was previously documented only for some ruminants^11,12^. As for arvicoline rodents, ruminants are also characterized by an increased rate of karyotype evolution among mammals.

In the evolution of the X chromosomes of modern species of voles a single case of potential convergence was identified, a convergent pericentric inversion in *C. gud* and *A. amphibius*. It is noteworthy that the number of convergent events recorded in the autosomes was significantly higher^20,21^.

The X chromosome of the putative ancestor of almost all species of the Microtina subtribe, except for *C. gud*, is characterized by a centromere shift. Centromere shifts in the X chromosomes have been found in other species. For example, the X chromosomes of elephant and humans differ by the position of the centromere but maintain the same gene order. A similar situation was observed in two species of the genus *Tokudaia*, Ryukian spiny mice^16^. It is also known that the X chromosome of the squirrel monkey (genus *Saimiri*) differs from human`s only in the formation of ENC^53^. Further, cladistic analysis shows that a reverse shift of the centromere back to its original position might have occurred in the evolutionary branch leading to the genus *Microtus*, specifically in *M. dogramacii*, *M. guentheri,* and *M. schidlovskii*. However, an alternative hypothesis is that multiple repeated events of convergence have also affected sex chromosomes in these species. It is impossible to rule out that centromere repositioning occurred repeatedly and independently in the phylogenetic lineages leading to the genera *Lasiopodomys, Alexandromys, Blanfordimys,* and *Terricola.*

The number of intrachromosomal rearrangements varies significantly in different branches of the vole phylogenetic tree. In general, rearrangements affecting the localization areas of probes A and B were observed in representatives of the basal branches, i.e. the tribes Ellobiini and Myodini, as well as in species *C. gud, A. amphibius,* and *M. rossiaemeridionalis*. For the remaining species of the Arvicolini tribe, the preservation of this segment in its ancestral form was shown.

Earlier, in the study of bird genomes, it was suggested that inversions are more often fixed in sex chromosomes than in autosomes^54^. Among eutherian mammals, there are several examples of significant rearrangement of sex chromosomes compared to autosomes^11,24^, although in general X chromosomes are remarkably conserved^3,4,7^. In case of arvicoline species we were unable to confirm or disprove that intrachromosomal rearrangements are more frequent in sex chromosomes than in autosomes. Firstly, the study of intrachromosomal rearrangements was carried out on the example of only three autosomal conservative segments of the ancestral karyotype, not on the entire autosomal set. Secondly, the uneven frequency of occurrence of rearrangements even in the three analyzed segments led us to suggest that the analysis of a larger number of segments could significantly change our ideas about the contribution of intrachromosomal rearrangements to the evolution of autosomal sets^20,21^. We found a higher occurrence of intrachromosomal rearrangements of the X chromosome only in the genus *Ellobius*^21^. In some species (*C. gud, B. afghanus*) intrachromosomal rearrangements in X chromosomes were found, while previous analyses showed that the autosomes were intact. The opposite situation was observed in three species of the genus *Terricola* and *L. gregalis* where conserved ancestral status of X chromosomes was accompanied by a great number of rearrangements in three previously analyzed segments of autosomes.

### The contribution of repeated sequences to the evolution of sex chromosomes

Conventional cytogenetic technique, such as C-banding, is able to detect and descript regions of accumulation of constitutive heterochromatin in karyotype^55^. Characteristically, constitutive heterochromatin consists largely of highly repetitive DNA. The use of AT-/GC-specific fluorochromes discovered great variability in the heterochromatin composition^56^. Simple repetitive sequences (e.g., microsatellites) are often accumulated in high copy numbers on the sex chromosomes in many taxa^57-59^, although the same repeats can be distributed throughout the genome in low copy numbers^55^. But in some species moderately repetitive sequences rather than highly repetitive DNA represent blocks of heterochromatin.

In voles, autosomal heterochromatin is mainly centromeric and contains dissimilar, repeated families in different species^50^. The blocks of constitutive heterochromatin on sex chromosomes are highly heterogeneous^52^ and also contain varying repeated DNA^50^. It was believed that heterochromatic variation does not appear to play a role in the speciation of arvicoline rodents^60^. But the results of this research and recently published studies indicate that the accumulation of repeated sequences could play a significant role in the evolution of X chromosomes of the voles^24^.

In most cases, our set of probes completely covered the entire X chromosome, however, for some species we encountered difficulties in analyzing the results. Some X chromosome regions were not hybridized by the probes, or, conversely, individual probes apparently had additional signals. We expected to get additional signals from probes C, D, and E because they partly overlapped the heterochromatic region of p-arm of *T. savii* X chromosome. Indeed, additional signals from probe C were observed on sex chromosomes of almost all species, but their localization did not always correlate to the distribution of heterochromatin.

Probe C marked the pericentromeric regions of all autosomes and sex chromosomes in the *T. savii* karyotype which might be due to a species-specific amplification and accumulation of repeats (Fig. 1e). The probe also slightly hybridized with pericentromeric regions of X chromosomes of all representatives of the genus *Terricola* and had a weak background signal in pericentromeric regions of X chromosomes of *M. arvalis* and *A. evoronensis* (Figs. 2e, 3). Within the genus *Terricola*, the size of hybridized areas varied greatly. C-banding shows that this variation in signal size is associated with the size of the heterochromatic regions (Fig. 3). In the karyotype of *T. savii*, the pericentromeric region of the X chromosome is C-positive, but no distinct blocks were found in the pericentromeric regions of karyotypes of the rest of the species listed above. Apparently, repeated sequences may be both species-specific and heterogeneous within the same chromosome^50^.

The pericentromeric regions of the X chromosomes of other species were not labeled with any of the probes, which may be explained by the fact that during the hybridization, repetitive sequences were suppressed using Cot DNA isolated from tissues of different species of voles (mainly, *Microtus*), or that there is little homology in pericentromeric repeats found in different species. However, it should be noted that three species (*M. dogramacii, M. guentheri, M. schidlovskii*), with large unlabeled pericentomeric regions (Fig. 3), belong to the same branch of the phylogenetic tree (Fig. 5). This may indicate the main role of accumulation of repeats in the evolution of sex chromosomes of these species. Moreover, C-banding did not reveal any large blocks of heterochromatin in the pericentromeric region of the X chromosomes of *M. dogramacii* (Fig. 3).

Previous research^26^ showed that *M. dogramacii* used here has an acrocentric X chromosome, but a metacentric X chromosome was described for this species by other authors^61^. However, this difference is not so surprising because interspecies chromosome polymorphism has been widely reported for voles, which affects both the centromere positions and the number of chromosomes (for example, for *A. mujanensis* in Lemskaya et al.^18^) and for *M. dogramacii* in Lemskaya et al.^26^).

Clear additional signals in the localization of probes D and E were detected only in *M. rossiaemeridionalis*, and their correspondence to the heterochromatic regions was established (Figs. 2b,d,3). There was a region on the q-arm of X chromosome of *T. daghestanicus* between signals from probes D and E, corresponding to a heterochromatic block (Figs. 2b,d,3). In this region we observed an additional signal from probe C, but there were no signals from probes D and E. This result may indicate similarity or convergence of repeated sequences in *T. savii* and *M. rossiaemeridionalis* and distinguishing them from other species used in this study.

Probe B provided an unusual result on chromosomes of *C. gud* and *A. amphibius*. Although the arrangement of the signals was the same for these species, in *C. gud* the additional signal was weaker than the main signal and corresponded to the dark heterochromatic region on the C-banded X chromosome but in *A. amphibius* both signals had the same intensity (Fig. 2c,d). This difference may be due to variations in the amount and accumulation of repeated sequences^46,48^.

In some cases, only cell cultures established for males were available for analysis. This allowed us to detect that probes C, D, and E provided signals on the Y chromosome of the species *A. evoronensis, A. mujanensis, B. juldaschi, M. guentheri, M. rossiaemeridionalis*, *M. schidlovskii,* and *L. gregalis* (Fig. S1). On the Y chromosome of *M. rutilis*, probe D provided a strong signal while signals of probes C and E were weak. No signals were found on the Y chromosome of *A. tuvinicus* and *C. gud.* In the case of the XX-male *E. talpinus*, no additional signals were observed on any other chromosomes. The results obtained are consistent with earlier studies in which the heteromorphism of X chromosome of the male *E. talpinus* was detected only in the analysis of meiosis^47^. The localization of the C, D, and E probes on the Y chromosomes of some vole species suggests the similarity of repeated sequences in their X and Y chromosomes. We did not find any relationship between the localization of probes on the Y chromosome and the synaptic or asynaptic behavior of sex chromosomes in meiosis in males^62^.

Recently it was shown that accumulation and expansion of microsatellites and DNA transposons might involve heterochromatinization and initiate sex chromosome differentiation in various taxa^59,63,64^. Thus, chromosomes having similar morphology and G-banding pattern can accumulate different repeated sequences in heterochromatic regions, i.e. repeated sequences can be species-specific and, conversely, variation in repeated sequences can give different variants of sex chromosomes in one species^22,50^. It was suggested that the karyotype of a common ancestor of modern arvicoline species contained varying repetitive families, and that descendants selectively amplified or deleted different repeats on different chromosomes^50^. This led to interspecific variability in the chromosomal distribution and number of copies of repeats. The result of the present work tested previously mentioned hypotheses on the particular lability of the arvicoline sex chromosomes in relation to C-band modification^60^ and also suggests a significant heterogeneity of the heterochromatic regions of the X chromosomes of voles^46-50,60^.

## Conclusions

Studies of the evolution of the genomes of non-mammalian species show that the euchromatic portion of the X chromosome is the fastest evolving by genomic rearrangements^65,66^, and sex chromosomes more often than autosomes drive speciation and hybrid incompatibilities^67^. Apparently in this case the evolution of repeated sequences has played a major role in incompatibilities, which could help to maintain reproductive isolation between species^68^. The same mechanisms may operate in mammalian species. In voles, we observe that evolution of the sex chromosomes was accompanied by multiple, not previously identified intrachromosomal rearrangements. As in the case of autosomes, para- and pericentric inversions and centromere shifts were common in the evolution of X chromosomes. Apparently identical types of rearrangements sometimes arose independently in different branches of the phylogenetic tree of voles. Unlike other taxa, it seems that the contribution of intrachromosomal rearrangements to the formation of karyotypes of the modern arvicoline species was approximately equivalent for the separate conservative segments of autosomes and X chromosomes. Moreover, the apparent diversity of X chromosomes of the voles by the presence, location, and size of heterochromatic blocks indicates that further study of intrachromosomal rearrangements in this taxon requires the study of repeated sequences in order to assess their contribution to the evolution of sex chromosomes.

## Supplementary information

Supplementary information.
