# Supplementary material for: Evolutionary rearrangements of X chromosomes in voles (Arvicolinae, Rodentia)
Source: Sci Rep. 2020 Aug 6;10:13235. doi: 10.1038/s41598-020-70226-4 (PMC7413345; doi:10.1038/s41598-020-70226-4)
Supplement: Supplementary file 1 — Supplementary information. [file 41598_2020_70226_MOESM1_ESM.pdf]

Evolutionary rearrangements of X chromosomes in voles (Arvicolinae, Rodentia)

Svetlana A. Romanenko[1*], Yulia E. Fedorova[1,2], Natalya A. Serdyukova[1], Marco Zaccaroni[3], Roscoe Stanyon[3], Alexander S. Graphodatsky[1]

[1] Institute of Molecular and Cellular Biology, SB RAS, Novosibirsk, Russia

[2] Novosibirsk State University, Novosibirsk, Russia

[3] Department of Biology, University of Florence, Florence, Italy

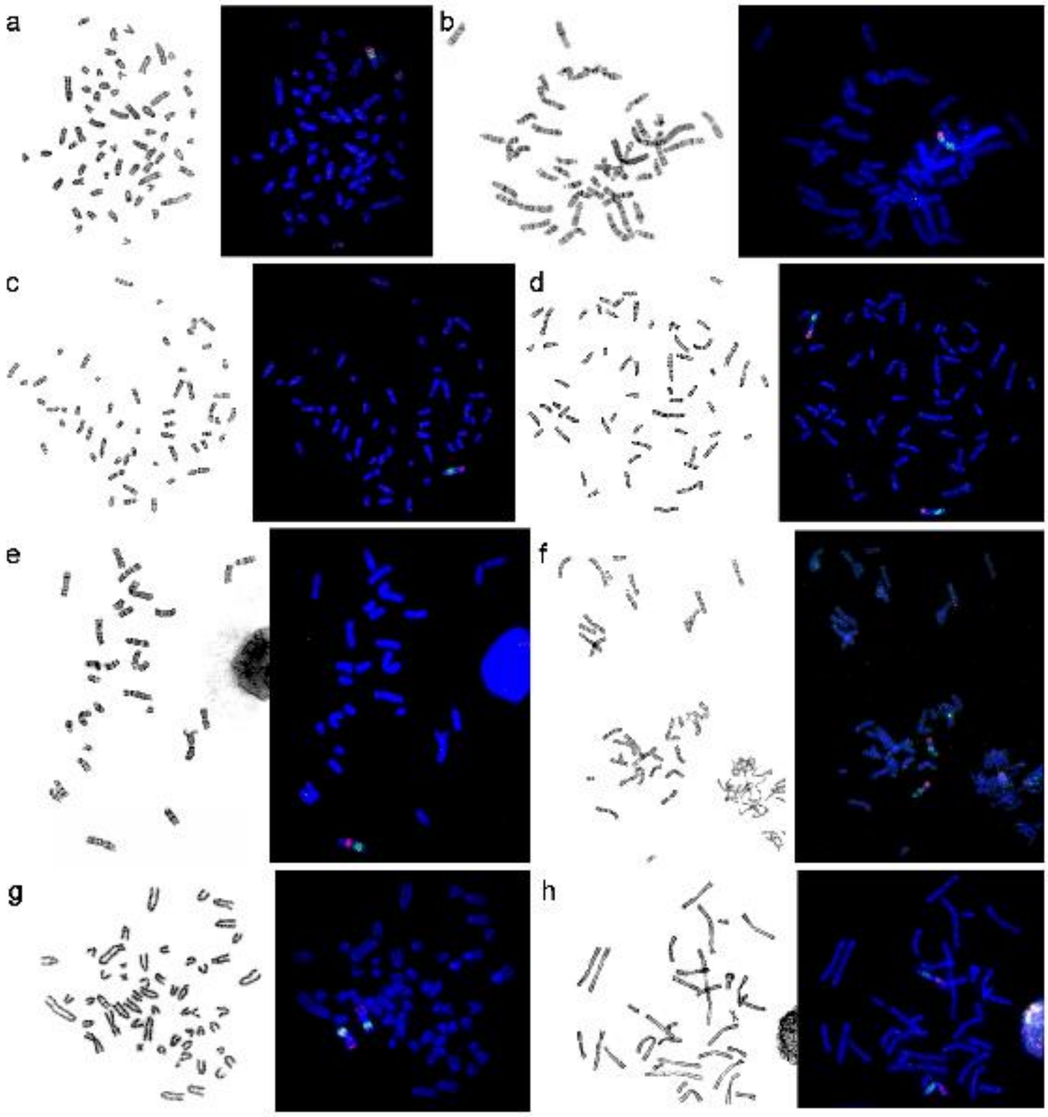

Figure S1A. Results of localization of probes A (red) and B (green) on the chromosomes of some species of voles: (**a**) – *M. schidlovskii*, (**b**) – *M. rossiaemeridionalis.* Results of localization of probes B (green) and E (red) on the chromosomes of some species of voles: (**c**) – *A. tuvinicus*, (**d**) – *B. afghanus*, (**e**) – *A. maximowiczii*, (**f**) – *A. amphibius*, (**g**) – *T. daghestanicus*, (**h**) – *L. brandtii*. GTG-banding is on the left.

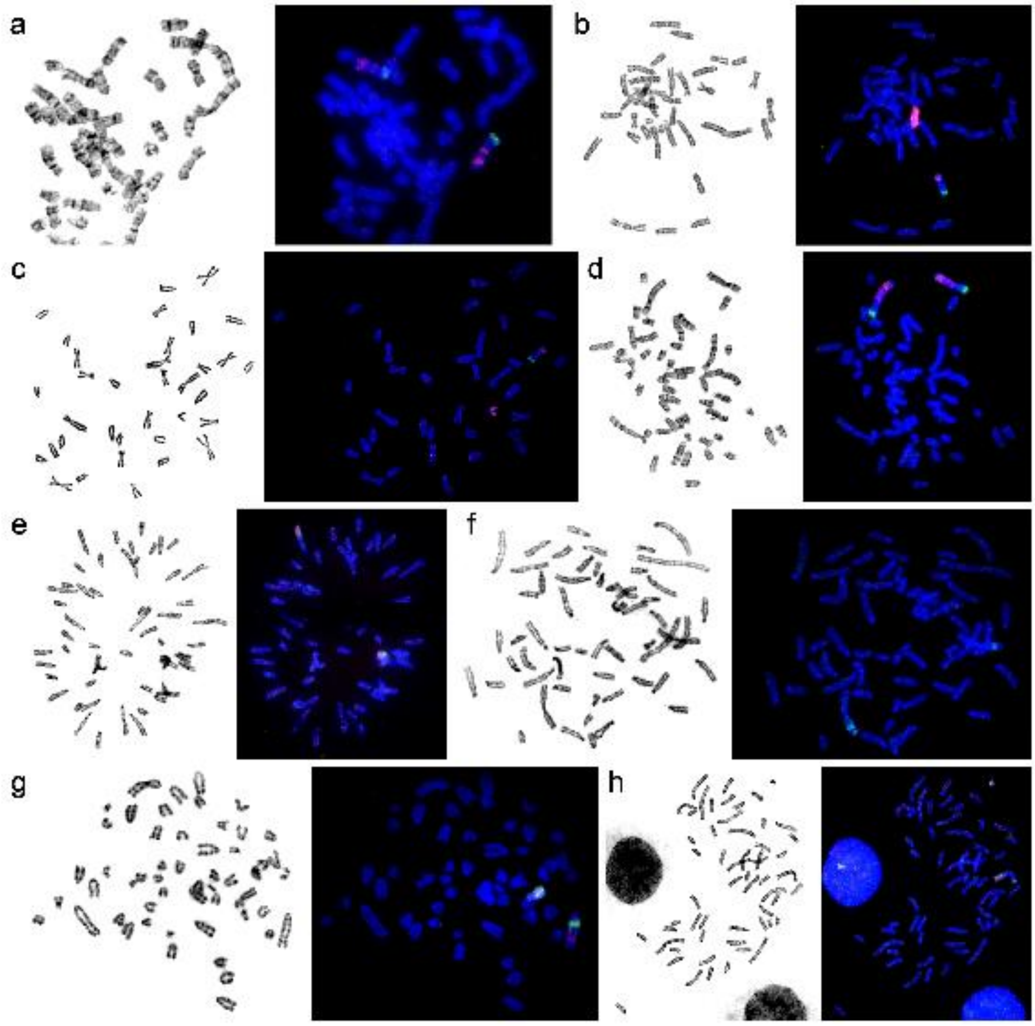

Figure S1B. Results of localization of probes A (green) and D (red) on the chromosomes of some species of voles: (**a**) – *A. maximowiczii*, (**b**) – *A. mujanensis,* (**c**) – *L. gregalis,* (**d**) – *T. majori*. (**e**) – localization of probes A (green) and B (red) on the chromosomes of *E. talpinus*. (**f**) – localization of probe C (green) on the chromosomes of *B. afghanus*. (**g**) – localization of probes E (green) and C (red) on the chromosomes of *B. juldashi*. (**h**) – localization of probes D (green) and E (red) on the chromosomes of *M. rutilus*. GTG-banding is on the left.
